# Editorial: Advances in brain functional network reconfiguration in psychosis

**DOI:** 10.3389/fpsyt.2025.1672185

**Published:** 2025-08-13

**Authors:** Chenfei Ye, Andreia V. Faria, Haoran Dou

**Affiliations:** ^1^ School of Biomedical Engineering, Harbin Institute of Technology, Shenzhen, China; ^2^ Department of Radiology, Johns Hopkins University School of Medicine, Baltimore, MD, United States; ^3^ Institute for Brain and Psychological Sciences, Sichuan Normal University, Chengdu, China

**Keywords:** network reorganization, brain functional connectivity, psychosis, TMS, acupuncture

Psychotic disorders, including schizophrenia, bipolar disorder with psychotic features, and related conditions, are characterized by profound disruptions in brain functional connectivity. These disruptions often manifest as aberrant reconfiguration of neural networks, affecting cognition, emotion regulation, and perception. The Research Topic “Advances in Brain Functional Network Reconfiguration in Psychosis” seeks to elucidate the mechanisms underlying these dynamic network changes, with a focus on neuroimaging evidence, clinical implications, and innovative therapeutic interventions. By integrating image markers from functional magnetic resonance imaging (fMRI), this Research Topic aims to advance our understanding of how brain networks adapt, or fail to adapt, in psychosis, ultimately informing novel diagnostic and treatment strategies. In a broader context, this work aligns with emerging paradigms in network neuroscience, where psychosis is viewed not as isolated regional deficits but as systemic imbalances in large-scale brain networks such as the default mode network (DMN), salience network, and central executive network (1). Such frameworks hold promise for personalized medicine, bridging basic research with clinical applications to mitigate the global burden of psychotic disorders.

This Research Topic brings together four original contributions that collectively highlight the multifaceted nature of brain network reconfiguration in psychosis and related conditions. Moving beyond a mere cataloging of findings, we emphasize how they interconnect to reveal patterns of neural plasticity, treatment-induced modulation, and methodological innovations.

The systematic review conducted by Lin et al. synthesizes neuroimaging evidence from 26 studies spanning 2014 to 2024, examining acupuncture’s impact on brain regions implicated in depressive disorders—a common comorbidity in psychosis. Drawing on data from over 1,100 participants, the review identifies consistent acupuncture-induced alterations in the cingulate gyrus, precuneus, insula, and prefrontal cortex, often correlating with Hamilton Rating Scale for Depression (HAMD) scores. These findings suggest that acupuncture may facilitate network reconfiguration in emotion regulation circuits, potentially extending to psychotic states where depressive symptoms exacerbate network instability. By adhering to rigorous standards like the Risk of Bias 2.0 (RoB 2) and Risk of Bias in Non-Randomized Studies of Interventions (ROBINS-I) tools, the authors underscore the need for standardized neuroimaging protocols to validate these effects. Building on this foundation, Sun et al. explore the neural correlates of electroacupuncture in modulating self-awareness and language-related networks in treating pediatric cerebral palsy, drawing parallels to psychosis-associated deficits in areas like Broca’s and Wernicke’s regions. This case study posits that acupuncture enhances connectivity within the anterior insula and medial temporal lobe—regions critical for self-referential processing, which are often impaired in cerebral palsy. This work contributes to the growing evidence base for acupuncture’s efficacy in neurodevelopmental and post-stroke conditions (2).

Complementing these reviews on neuromodulation, Zhang et al. present an empirical study investigating the effects of individual target-transcranial magnetic stimulation (IT-TBS) on brain network topology in major depressive disorder with suicidal ideation. Following a 5-day IT-TMS protocol (Stanford Neuromodulation Therapy), patients exhibited reductions in suicidal ideation and depression symptoms. Crucially, observed inefficiencies in small-world network properties and aberrant functional segregation shifted towards a normalization pattern resembling healthy controls post-treatment. A specific decrease in functional connectivity between the right insula and left anterior cingulate gyrus specifically correlated with clinical improvement. This suggests that IT-TMS alleviates symptoms by reducing hyperactivity within the salience network, potentially by optimizing the trade-off between metabolic costs and network integration efficiency.

Similarly, Zhang et al. apply graph theory analysis to examine brain functional network alterations in 50 schizophrenia patients with persistent auditory verbal hallucinations, focusing on the effects of repetitive transcranial magnetic stimulation (rTMS) over the left temporoparietal junction (TPJ). The disrupted small-world properties reduced functional segregation, and impaired connectivity in language and auditory networks observed in schizophrenia patients showed renormalization after rTMS treatment, with improvements in global efficiency, clustering coefficients, and modularity correlating with reductions in Positive and Negative Syndrome Scale (PANSS) scores for hallucinations. This work underscores the utility of graph-theoretical approaches in quantifying psychosis-related dysconnectivity and evaluating TMS-induced network reconfiguration, providing evidence for targeted interventions in treatment-resistant symptoms.

Together, these articles illuminate the potential of neuroimaging to map psychosis-related network dynamics, while advocating for integrative approaches like acupuncture and TMS to induce beneficial reconfigurations. They also identify critical gaps, particularly the need for longitudinal designs and larger cohorts to address biases inherent in non-randomized trials. Within the broader landscape of psychiatric research, this Research Topic contributes to a paradigm shift towards network-based models, fostering interdisciplinary collaboration to translate these insights into clinical practice. These contributions emphasize the centrality of adaptive brain network function in recovery and resilience. Looking ahead, future studies should explore multimodal neuromodulation strategies (e.g., combining acupuncture and TMS) to investigate common mechanisms of brain network regulation across psychiatric disorders (3).
